# Reversible encephalitis-like episodes in fragile X-associated tremor/ataxia syndrome: a case report

**DOI:** 10.1186/s12883-024-03641-z

**Published:** 2024-05-07

**Authors:** Shaoping Zhong, Jianying Liu, Yangye Lian, Binbin Zhou, Xin Wang, Jing Ding

**Affiliations:** 1 Department of Neurology, Zhongshan Hospital, Fudan University, 180 Fenglin Road, Shanghai, 200032 China; 2 The State Key Laboratory of Medical Neurobiology and MOE Frontiers Center for Brain Science, Institutes of Brain Science, Fudan University, Shanghai, China; 3 CAS Center for Excellence in Brain Science and Intelligence Technology, Shanghai, China

**Keywords:** Fragile X-associated tremor/ataxia syndrome, Encephalitis-like episode, Neuronal intranuclear inclusion disease, Biopsy, Trinucleotide repeat expansion

## Abstract

**Background:**

Fragile X-associated tremor/ataxia syndrome (FXTAS) is a neurodegenerative disorder caused by CGG repeat expansion of *FMR1* gene. Both FXTAS and neuronal intranuclear inclusion disease (NIID) belong to polyglycine diseases and present similar clinical, radiological, and pathological features, making it difficult to distinguish these diseases. Reversible encephalitis-like attacks are often observed in NIID. It is unclear whether they are presented in FXTAS and can be used for differential diagnosis of NIID and FXTAS.

**Case presentation:**

A 63-year-old Chinese male with late-onset gait disturbance, cognitive decline, and reversible attacks of fever, consciousness impairment, dizziness, vomiting, and urinary incontinence underwent neurological assessment and examinations, including laboratory tests, electroencephalogram test, imaging, skin biopsy, and genetic test. Brain MRI showed T2 hyperintensities in middle cerebellar peduncle and cerebrum, in addition to cerebellar atrophy and DWI hyperintensities along the corticomedullary junction. Lesions in the brainstem were observed. Skin biopsy showed p62-positive intranuclear inclusions. The possibilities of hypoglycemia, lactic acidosis, epileptic seizures, and cerebrovascular attacks were excluded. Genetic analysis revealed CGG repeat expansion in *FMR1* gene, and the number of repeats was 111. The patient was finally diagnosed as FXTAS. He received supportive treatment as well as symptomatic treatment during hospitalization. His encephalitic symptoms were completely relieved within one week.

**Conclusions:**

This is a detailed report of a case of FXTAS with reversible encephalitis-like episodes. This report provides new information for the possible and rare features of FXTAS, highlighting that encephalitis-like episodes are common in polyglycine diseases and unable to be used for differential diagnosis.

## Background

Fragile X-associated tremor/ataxia syndrome (FXTAS) is a late-onset neurodegenerative disorder characterized by intention tremor and/or cerebellar ataxia due to CGG repeat expansion in the premutation range (55–200) of *FMR1* gene [1]. FXTAS is often initiated insidiously with intension tremor in subjects above 50 years. Progressive cerebellar ataxia is another core feature of FXTAS, being the only clinical symptom in about 20% of these patients. Additional symptoms include cognitive impairment, peripheral neuropathy, and autonomic dysfunction like hypotension, erectile dysfunction, and incontinence [2]. Characteristic p62- and ubiquitin-positive intranuclear inclusions and white matter lesions in magnetic resonance imaging (MRI) are useful for the diagnosis of FXTAS [3].

An important differential diagnosis for FXTAS is adult-onset neuronal intranuclear inclusion disease (NIID), a neurodegenerative disorder caused by CGG repeat expansion in *NOTCH2NLC* gene [4, 5]. Recent studies proposed that both FXTAS and NIID belong to polyglycine (polyG) diseases, a group of CGG repeat expansion disorders sharing clinical, radiological, and pathological similarities as well as common molecular mechanisms [6, 7]. Encephalitis-like episodes are a frequently observed and highly suggestive of NIID [8]. However, it is unclear whether such a manifestation presented in FXTAS and can be used for differential diagnosis of FXTAS and NIID.

We report a case of FXTAS with multiple reversible encephalitis-like attacks, who experienced two episodes with fever, consciousness impairment, dizziness, and vomiting followed by complete remission of these symptoms. This report describes a rare clinical manifestation of FXTAS, which may be helpful in updating our understanding of FXTAS clinical features.

## Case presentation

A 63-year-old right-handed Chinese male of Han nationality, with a history of well-controlled type 2 diabetes mellitus for 1 year, hypertension for 15 years, and ascending aorta aneurysm for 6 years, presented with progressive and untreated gait disturbance since his 50s. His regular medication includes metformin, candesartan, aspirin and atorvastatin. He was admitted to hospital with an acute neurological episode, complaining of dizziness, vomiting, urinary incontinence, fever, and subsequent consciousness disturbance in the previous 6 h. He had experienced a similar attack one year ago, lasting for several days before the full-recovery. Upon admission, he was in light coma with Glasgow Coma Scale (GCS) score [9] of 8/15. The physical examination revealed a temperature of 38.5 °C, a pulse of 95 beats/min, breath rate of 16 times/min, and a blood pressure of 136/81 mmHg. Neurological examination revealed no abnormalities in cranial nerve function except miosis, with bilateral pupil diameter of 1.5 mm. The bilateral knee reflex, Achilles tendon reflex, biceps reflexes, and triceps reflexes were diminished. The bilateral plantar responses were flexor. His limbs showed flexion response to pinprick stimulation. He had no meningeal irritation signs. Laboratory tests revealed normal random blood glucose (9.8 mmol/L) and normal blood lactate (2.09 mmol/L), excluding possibilities of hypoglycemia and lactic acidosis. Routine blood tests revealed the patient’s leukocyte count was 9.65 × 10^9^/L (the normal range is 3.50–9.50 × 10^9^/L), and his neutrophil percentage was 82.2% (the normal range is 40.0–75.0%). His C-reactive protein level was 50.2 mg/L (the normal range is 0.0–3.0 mg/L), and he had normal procalcitonin (0.48 ng/mL). Cardiac marker tests revealed slightly elevated level of seral NT-proBNP (644 pg/mL) but normal high-sensitive cardiac troponin T (0.017 ng/mL). Electrocardiography and blood tests including electrolytes, blood ammonia, hepatorenal function, thyroid hormones, ceruloplasmin, and autoantibodies showed no abnormalities. He had normal titer of antibodies against viruses such as HIV, herpes simplex virus, cytomegalovirus, and Epstein-Barr virus, as well as Treponema pallidum. Computed tomography (CT) revealed leukoaraiosis in the brain, chronic inflammation in both lungs, aneurysmal dilatation of the ascending aorta, coronary atherosclerosis, and cardiomegaly. Aortic dissection was excluded by computed tomography angiography (CTA). However, lumbar puncture was not conducted because of the unwillingness of the patient’s guardian.

**Fig. 1:**
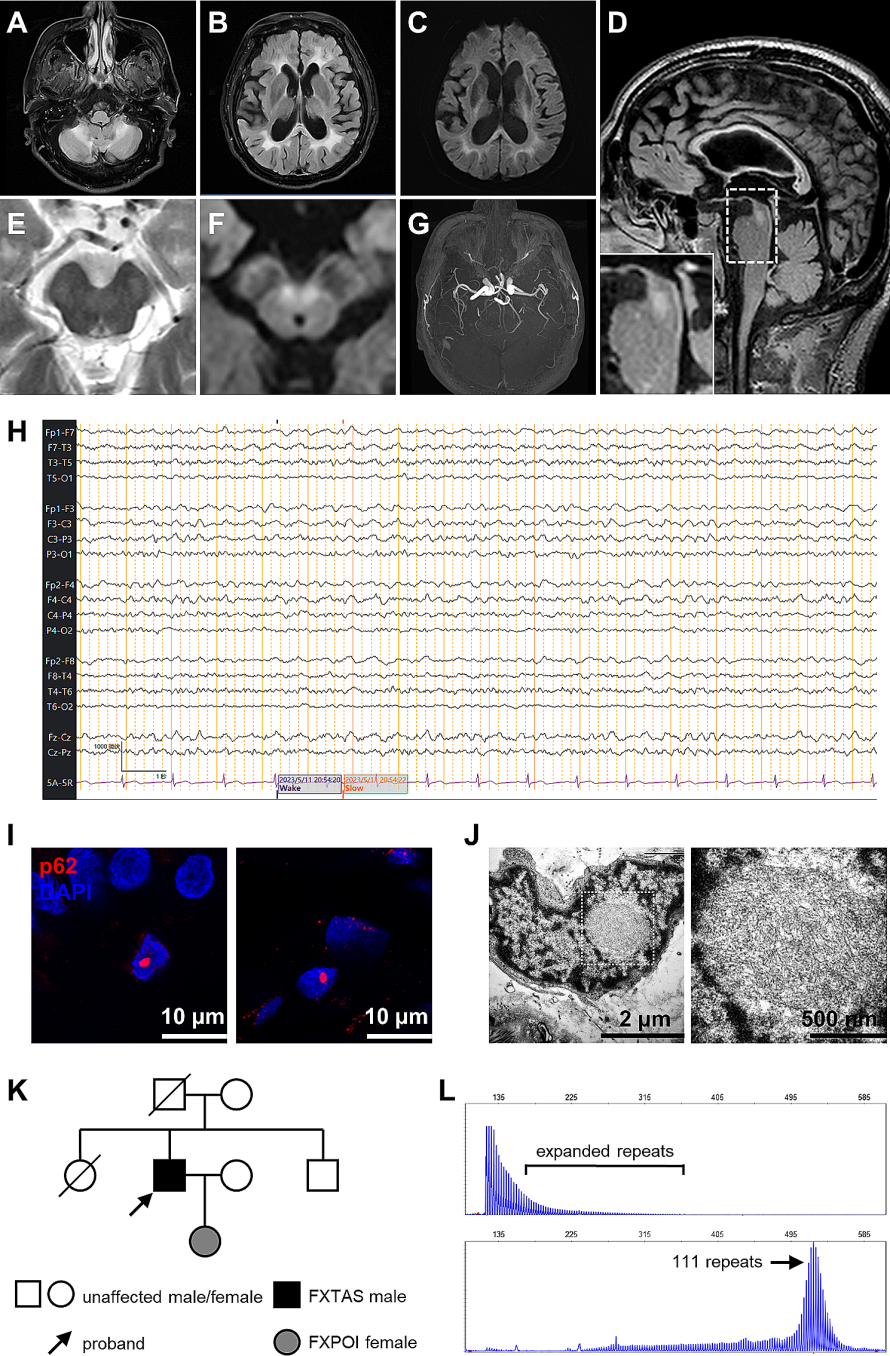
Radiological, electrophysiological, genetic and histopathological findings of an FXTAS patient. (**A-G**) Head MRI findings. Axial T2-FLAIR image showing increased signal intensity in the cerebellum (MCP sign; **A**) and cerebral white matter (**B**). Axial DWI images showing hyperintensities along the corticomedullary junction (Subcortical lace sign; **C**). Lesions in the midbrain are shown by the sagittal T2-FLAIR image (**D**), axial T2 image (**E**) and axial DWI image (**F**). An expanded view of the area in the white box is shown on the lower left of D. (**H**) EEG showed general theta waves without epileptic waves. (**I**) Immunofluorescent findings of the skin biopsy specimen. P62-positive intranuclear inclusions in fibroblasts (left) and adipocytes (right) are shown. (**J**) Transmission electron microscope images of an intranuclear inclusions in the patient. The right panel is an expanded view of the white box area in the left panel, showing electron-dense filaments inside the intranuclear inclusion. (**K**) Pedigree diagram of the patient. FXPOI, Fragile X-associated primary ovarian insufficiency. (**L**) Genetic analysis of *FMR1* gene. Repeat-primed polymerase chain reaction (RP-PCR) showed repeat expansion (upper panel). The number of CGG repeats was calculated by fragment analysis using PCR, and the patient had 111 repeats (lower panel)

On the 3rd day after admission, his consciousness gradually recovered, allowing us to perform further examinations. He showed limb ataxia but not tremor. His gait was wide-based and ataxic. Muscle weakness was not observed. Cognitive scales were assessed, with Montreal Cognitive Assessment (MoCA) [10] score of 22/30. Magnetic resonance imaging (MRI) revealed abnormal signals in the white matter region of the brain, especially in the middle cerebellar peduncle (MCP sign) (Fig. 1A) and cerebral deep white matter (Fig. 1B), in addition to brain atrophy and diffusion-weighted imaging (DWI) hyperintensities along the corticomedullary junction (subcortical lace sign) (Fig. 1C). T2 and DWI hyperintensities in the midbrain were also noted (Fig. 1D-F). Magnetic resonance angiography (MRA) demonstrated decreased flow but no intracranial aortic stenosis or occlusion (Fig. 1G). On the 7th day after admission, he received bedside video electroencephalogram (EEG) for 15 h. EEG revealed generalized slow waves, with Medium-to-high amplitude (1.7–7.0 Hz, 30–100 µV) and scattered or paroxysmal distribution, in the awakening periods, whereas no epileptic waves were detected (Fig. 1H). Holter electrocardiography showed no abnormalities.

To identify the etiology of this case, we performed skin biopsy on the right leg. Hematoxylin-eosin staining revealed eosinophilic spherical inclusion bodies in the nuclei of some fibroblasts and adipocytes. Immunofluorescence staining showed these intranuclear inclusions were p62-positive (Fig. 1I). Observed by electron microscope, intranuclear inclusions which were membraneless and composed by disorderly-oriented and electron-dense fibrils (Fig. 1J). He denied a family history of neurological disease, but his 32-year-old daughter suffers from amenorrhea since her 20s and was diagnosed as premature ovarian failure (Fig. 1K). Genetic tests using repeat-primed PCR and GC-rich PCR revealed an expansion of 111 CGG repeats in *FMR1* gene (Fig. 1L) whereas negative results for *NOTCH2NLC*, *LRP12*, *GIPC1*, and *RILPL1* genes.

During hospitalization, the patient was given symptomatic treatment such as anti-dizziness and anti-vomiting treatment as well as supportive treatment such as nutrition support therapy and vitamin B and C supplements. Antihypertensive treatment, Aspirin, and atorvastatin were also given by routine. His symptoms in the encephalitic attack, including dizziness, vomiting, urinary incontinence, fever, and clouding of consciousness, were completely relieved within one week. After discharge, follow-ups at 3 and 6 months revealed the patient still had gait disturbance, but no longer had encephalitis-like symptoms.

## Discussion and conclusions

This case study describes a patient with FXTAS who presented with two acute episodes within one year. The patient met the diagnostic criteria for “definite” FXTAS, based on his clinical manifestations (ataxia and cognitive decline), characteristic radiological features (MCP sign and subcortical lace sign), typical intranuclear inclusions, and *FMR1* gene premutation [3]. FXTAS is a special neurodegenerative disorder with complicated manifestations such as intention tremor, cerebellar ataxia, cognitive impairment, and autonomic dysfunction. The similarities of FXTAS and NIID make differentiation diagnosis difficult without the help of genetic analysis [4]. We have excluded the possibility of NIID in this case because the number of *NOTCH2NLC* CGG repeats was within the normal range (11 and 12 repeats, respectively).

To our knowledge, this is the first case report of FXTAS presenting with reversible encephalitis-like attacks. Atypical manifestations, such as Parkinsonism, involuntary movements (e.g. generalized chorea), and myogenic lesion (e.g. inclusion body myositis), have been noted in some FXTAS patients [11, 12]. However, paroxysmal symptoms that are frequently seen in NIID patients, including encephalitis-like attacks [13, 14], stroke-like attacks [15], migraine-like attacks [16], and epileptic episodes [17], are rarely documented in FXTAS, except for a previous report about an Italian FXTAS female with stroke-like attacks of acute vomiting, dysarthria, dizziness, quadriparesis, and consciousness impairment [18]. Presenting in more than 20% of NIID cases, encephalitis-like attacks are a unusual manifestation characterized by subacute onset of fever, headache, vomiting and disturbance of consciousness without the evidence of bacterial or viral meningitis and encephalitis [8, 13, 15, 19]. In most NIID cases, encephalitis-like attacks were recovered in a few weeks, leaving little or none residual symptoms. The symptoms and disease course of this patient were similar with those attacks in NIID. One of the limitations of this study is that we cannot entirely rule out the possibility of infectious encephalitis in this case due to the absence of lumbar puncture and cerebrospinal fluid analyses. However, the lack of meningeal irritation signs, radiological alterations related to intracranial infection, and antibodies against common encephalitic pathogens did not support an infectious cause. The possibilities of other disorders that may contribute to his paroxysmal symptoms, such as cerebrovascular events, epilepsy, and lactic acidosis, were excluded by auxiliary examinations. Thus, the diagnose of encephalitis-like episode was considered. This suggests that encephalitis-like episodes are not confined to NIID but also presented in FXTAS. Therefore, such attacks cannot be used for differential diagnosis of FXTAS and NIID.

The pathogenesis of encephalitis-like episodes in FXTAS and NIID remains undetermined. Some studies have found dynamic perfusion abnormality and focal brain edema with gadolinium enhancement in NIID cases with neurologic attacks [19–21], suggesting cerebral blood flow changes may contribute to these attacks. A limitation of the present study is that we were unable to exam the perfusion changes at the beginning of the attack. It remains to be investigated whether cerebrovascular dysfunction exists in FXTAS with paroxysmal symptoms. In addition, we noted brainstem lesions through MRI in this case. In the other FXTAS case, Orsucci et al. also observed obvious brainstem lesions, and proposed that brainstem dysfunction can be account for the vertebrobasilar stroke-like attack of the case [18]. However, it is reported that intensity abnormalities of brainstem are as common as that of corpus callosum splenium and the cerebral deep white matter in FXTAS [22]. Thus, the etiology and prevention of encephalitic attacks in FXTAS and NIID needs to be further studied.

Similar to other neurodegenerative diseases, disease-modifying treatment for FXTAS is still lacking. Only supportive therapies and symptomatic treatments are available currently. It was reported that steroid pulse therapy may be effective to reduce brain edema and improve encephalitic symptoms in NIID in the short term [19], while whether this therapy can be applicable to the encephalitic attacks in FXTAS is unknown.

It should be noted that CGG repeat expansion in *FMR1* gene is responsible for several diseases, including fragile X syndrome, a common cause of inherited intellectual disability that is caused by much larger expansions (full mutation; >200 CGG repeats), and FXTAS as well as fragile X-associated primary ovarian insufficiency (FXPOI), which are caused by *FMR1* premutation [23]. Based on X-linked inheritance, we suppose that the patient’s daughter also carries *FMR1* premutation and she is most likely an individual of FXPOI, although genetic diagnose was not obtained because of her unwillingness to receive genetic test.

In summary, we present a case of FXTAS with reversible encephalitis-like episodes, which has not been reported previously. This report increases our recognition of the possible and rare features of FXTAS, and highlights paroxysmal symptoms are common in polyG diseases. Clinicians should consider FXTAS as a differential diagnosis for undetermined etiology of encephalitis syndrome in a patient with leukoencephalopathy.

## Data Availability

No datasets were generated or analysed during the current study.

## Abbreviations

CT: Cranial computed tomography
CTA: Computed tomography angiography
DWI: Diffusion-weighted imaging
EEG: Electroencephalogram
FMR1: Fragile X mental retardation 1
FXTAS: Fragile X-related tremor/ataxia syndrome
FXPOI: Fragile X-associated primary ovarian insufficiency
GC-PCR: GC-rich PCR
MCP: Middle cerebellar peduncle
MRI: Magnetic resonance imaging
NIID: Neuronal intranuclear inclusion disease
NOTCH2NLC: Notch 2 N-terminal like C
NT-proBNP: N-terminal pro brain natriuretic peptide
polyG: Polyglycine
RP-PCR: Repeat-primed PCR
